# Robot assisted training for the upper limb after stroke (RATULS): a multicentre randomised controlled trial

**DOI:** 10.1016/S0140-6736(19)31055-4

**Published:** 2019-07-06

**Authors:** Helen Rodgers, Helen Bosomworth, Hermano I Krebs, Frederike van Wijck, Denise Howel, Nina Wilson, Lydia Aird, Natasha Alvarado, Sreeman Andole, David L Cohen, Jesse Dawson, Cristina Fernandez-Garcia, Tracy Finch, Gary A Ford, Richard Francis, Steven Hogg, Niall Hughes, Christopher I Price, Laura Ternent, Duncan L Turner, Luke Vale, Scott Wilkes, Lisa Shaw

**Affiliations:** a Stroke Research Group, Institute of Neuroscience, Newcastle University, Newcastle upon Tyne, UK; b Stroke Northumbria, Northumbria Healthcare NHS Foundation Trust, North Tyneside, UK; c Newcastle Hospitals NHS Foundation Trust, Newcastle upon Tyne, UK; d Massachusetts Institute of Technology, Cambridge, MA, USA; e School of Health and Life Sciences, Glasgow Caledonian University, Glasgow, UK; f Institute of Health and Society, Newcastle University, Newcastle upon Tyne, UK; g School of Healthcare, University of Leeds, Leeds, UK; h Barking, Havering and Redbridge University Hospitals NHS Trust, Romford, UK; i London North West Healthcare NHS Trust, Northwick Park Hospital, Harrow, UK; j Institute of Cardiovascular and Medical Sciences, University of Glasgow, Glasgow, UK; k Nursing, Midwifery and Health, Northumbria University, Newcastle upon Tyne, UK; l Medical Sciences Division, University of Oxford and Oxford University Hospitals NHS Foundation Trust, Oxford, UK; m NHS Greater Glasgow and Clyde, Glasgow, UK; n School of Health, Sport and Bioscience, University of East London, London, UK; o School of Medicine, University of Sunderland, Sunderland, UK

## Abstract

**Background:**

Loss of arm function is a common problem after stroke. Robot-assisted training might improve arm function and activities of daily living. We compared the clinical effectiveness of robot-assisted training using the MIT-Manus robotic gym with an enhanced upper limb therapy (EULT) programme based on repetitive functional task practice and with usual care.

**Methods:**

RATULS was a pragmatic, multicentre, randomised controlled trial done at four UK centres. Stroke patients aged at least 18 years with moderate or severe upper limb functional limitation, between 1 week and 5 years after their first stroke, were randomly assigned (1:1:1) to receive robot-assisted training, EULT, or usual care. Robot-assisted training and EULT were provided for 45 min, three times per week for 12 weeks. Randomisation was internet-based using permuted block sequences. Treatment allocation was masked from outcome assessors but not from participants or therapists. The primary outcome was upper limb function success (defined using the Action Research Arm Test [ARAT]) at 3 months. Analyses were done on an intention-to-treat basis. This study is registered with the ISRCTN registry, number ISRCTN69371850.

**Findings:**

Between April 14, 2014, and April 30, 2018, 770 participants were enrolled and randomly assigned to either robot-assisted training (n=257), EULT (n=259), or usual care (n=254). The primary outcome of ARAT success was achieved by 103 (44%) of 232 patients in the robot-assisted training group, 118 (50%) of 234 in the EULT group, and 85 (42%) of 203 in the usual care group. Compared with usual care, robot-assisted training (adjusted odds ratio [aOR] 1·17 [98·3% CI 0·70–1·96]) and EULT (aOR 1·51 [0·90–2·51]) did not improve upper limb function; the effects of robot-assisted training did not differ from EULT (aOR 0·78 [0·48–1·27]). More participants in the robot-assisted training group (39 [15%] of 257) and EULT group (33 [13%] of 259) had serious adverse events than in the usual care group (20 [8%] of 254), but none were attributable to the intervention.

**Interpretation:**

Robot-assisted training and EULT did not improve upper limb function after stroke compared with usual care for patients with moderate or severe upper limb functional limitation. These results do not support the use of robot-assisted training as provided in this trial in routine clinical practice.

**Funding:**

National Institute for Health Research Health Technology Assessment Programme.

## Introduction

Upper limb problems commonly occur after a stroke, comprising loss of movement, coordination, sensation, and dexterity, which lead to difficulties with activities of daily living (ADL) such as washing and dressing. About 80% of people with acute stroke have upper limb motor impairment, and of those with reduced arm function early after stroke, 50% still have problems after 4 years.^1^ The strongest predictor of recovery is severity of initial neurological deficit; patients with severe initial upper limb impairment are unlikely to recover arm function, with clear impact upon their quality of life. Patients report that loss of arm function is one of the most distressing long-term consequences of stroke. Improving upper limb function has been identified as a top ten research priority by stroke survivors, carers, and clinicians.^2^

How to optimise stroke patients' upper limb recovery is unclear. Systematic reviews of therapy interventions suggest that patients benefit from therapy programmes in which they practise tasks directly rather than from interventions that focus on impairments.^3^, ^4^ Intensity of therapy is also important; a Cochrane overview^4^ of systematic reviews found moderate quality Grading of Recommendations, Assessment, Development and Evaluations evidence that arm function after a stroke can be improved by the provision of at least 20 h of additional repetitive task training.

Robot-assisted arm training has shown promise for improving ADL, arm function, and arm muscle strength after stroke.^5^, ^6^ However, studies vary in patient characteristics, device used, duration and amount of training, control group, and outcome measures used. The benefits of robot-assisted arm training over conventional therapy of the same frequency and duration have not been shown.^7^

**Research in context**

**Evidence before this study**

Robot-assisted training enables stroke patients with moderate or severe upper limb impairment to perform repetitive tasks in a highly consistent manner, tailored to their motor abilities. A 2018 Cochrane review (45 trials, 1619 participants) reported that electromechanical and robot-assisted arm training improved arm function (standardised mean difference [SMD] 0·32 [95% CI 0·18–0·46]) and activities of daily living (ADL) scores (0·31 [0·09–0·50]). The clinical importance of these differences is unclear and the results should be interpreted with caution because of study heterogeneity and variation in the quality of the included studies. Another review found no difference in any of these outcomes when robot-assisted training was compared with the same duration or intensity of conventional therapy. Theories of neuroplasticity and motor learning support an approach to rehabilitation based on repetitive practice of functional tasks. A 2016 Cochrane review (33 trials, 1853 participants) found that repetitive functional task practice for patients who had a stroke was associated with improved arm function (SMD 0·25 [95% CI 0·01–0·49]), hand function (0·25 [0·00–0·51]), and ADL (0·28 [0·10–0·45]). Again, the clinical importance of these findings is unclear.

**Added value of this study**

RATULS is the first multicentre trial with adequate statistical power to compare robot-assisted training with both an evidence-based therapy programme of the same frequency and duration (45 min face-to-face therapy, three times per week, for 12 weeks) and usual care. We found no significant difference in our primary outcome of upper limb function at 3 months between stroke patients treated with robot-assisted training, an enhanced upper limb therapy programme (EULT), or usual care. Although some improvements for robot-assisted training were observed in upper limb impairment compared with usual care, these did not translate into improvement in upper limb function or ADL. EULT resulted in less upper limb impairment, better mobility, and better ADL at 3 months compared with usual care. Furthermore, EULT was superior to robot-assisted training for ADL at 3 months.

**Implications of all the available evidence**

The results of the RATULS trial do not support the routine use of robot-assisted training (MIT-Manus robotic gym) for patients with moderate or severe upper limb functional limitation resulting from stroke. Our findings will inform guidelines about the use of robot-assisted training and EULT in clinical practice. Further research is needed to find ways to translate the improvements in upper limb impairment seen with robot-assisted training into improvements in upper limb function and ADL. This might involve combining robot-assisted training with more functionally orientated therapy strategies. The RATULS trial provides evidence of the potential benefit of EULT, although as delivered in this trial, it is unlikely to be cost-effective. Innovations to make enhanced rehabilitation programmes more clinically effective and cost-effective are needed.

This randomised controlled trial (RCT) aimed to establish whether robot-assisted training improved upper limb function after a stroke compared with an enhanced upper limb therapy (EULT) programme of the same frequency and duration and usual care alone. Robot-assisted training and EULT involved repetitive task practice and were provided in addition to usual care.

## Methods

### Study design

RATULS was a three-group, pragmatic, multicentre RCT done at four National Health Service (NHS) centres in the UK. Each centre comprised a stroke service in an NHS hospital with an MIT-Manus robotic gym system (InMotion commercial version, Interactive Motion Technologies, Watertown, MA, USA), plus stroke services in adjacent NHS Trusts and community services. The trial protocol was approved by National Research Ethics Committee Sunderland (reference 13/NE/0274) and has been published.^8^

### Participants

Study participants were adults (age ≥18 years) with moderate or severe upper limb functional limitation (Action Research Arm Test [ARAT] score 0–39)^9^ as a result of first-ever stroke that had occurred between 1 week and 5 years before randomisation. Exclusion criteria were other notable impairment in the upper limb affected by stroke; other diagnosis that might interfere with rehabilitation or outcome assessments; previous use of the robotic gym system or other arm rehabilitation robot; participation in another upper limb rehabilitation trial; and previous enrolment in this study. Participants were recruited from stroke units, outpatient clinics, day hospitals, community rehabilitation services, local stroke clubs, and primary care. All participants provided written informed consent.

### Randomisation and masking

Randomisation was done through a central independent web-based service hosted by Newcastle University Clinical Trials Unit. Participants were randomly assigned 1:1:1 to receive robot-assisted training, an EULT programme, or usual care using permuted block sequences stratified according to centre, time since stroke, and severity of upper limb functional limitation (ARAT score).^9^ The sequences were prepared by an independent statistician before the start of enrolment. Outcome data were intended to be collected by a masked researcher and any unmasking was recorded. Because of the nature of the intervention, patients and therapists could not be masked to the allocated treatment.

### Procedures

Robot-assisted training and EULT programmes were delivered at the same frequency and duration: 45 min of face-to-face therapy, three times per week for 12 weeks. The same therapists and therapy assistants delivered both interventions at each centre. A detailed description of robot-assisted training and EULT using the Template for Intervention Description and Replication checklist is available.^8^ Robot-assisted training and EULT were delivered in addition to usual post-stroke care.

The robot-assisted training programme integrated training with all three modules of the MIT-Manus robotic gym (shoulder–elbow module, wrist module, hand module integrated on to the shoulder–elbow module). The EULT programme was designed to reflect best practice using repetitive functional task practice to work towards participant-centred goals.^10^, ^11^ Therapists recorded data on the content of EULT sessions; the MIT-Manus robotic gym recorded data on the robot-assisted training sessions content.

Participants assigned to usual care received usual NHS care, which was provided by their local clinical service. The English national quality standard is that patients with stroke should be offered a minimum of 45 min of each appropriate therapy that is required, for a minimum of 5 days per week, at a level that enables the patient to meet their rehabilitation goals for as long as they are continuing to benefit from therapy and as long as they are able to tolerate it. Many stroke units achieve this target for physiotherapy and occupational therapy, but considerable variation exists in service provision after discharge.^12^ Participants in all three groups received an arm rehabilitation therapy log to record any upper limb rehabilitation received during the trial and any self-practice arm exercises done. The schedule of enrolment, interventions, and assessments can be found in the protocol.^8^

Assessments included the ARAT,^9^, ^13^ which assesses upper limb function by scoring the ability to complete a range of functional tasks within four subscales (grasp, grip, pinch, and gross movement), with a total score from zero (no function) to 57 (normal function); Fugl–Meyer assessment (FMA);^13^, ^14^ Barthel ADL Index;^15^ Stroke Impact Scale (SIS), version 3.0;^16^ and measurement of upper limb pain on a numerical rating scale (score 0–10). For all the scores except the upper limb pain scale, a higher score denotes better performance. The total upper extremity FMA scale assesses upper limb impairment by incorporating the motor, sensory, range of motion, and joint pain subscales (score 0–126). The FMA motor subscale is frequently used as a primary outcome in trials of robot-assisted training (score 0–66). The Barthel ADL Index consists of ten activities, together scored from 0 to 20. The SIS is a self-completion, stroke-specific questionnaire to measure quality of life. There are nine dimensions (strength, hand function, mobility, ADL, emotion, memory, communication, social participation, and stroke recovery). The dimension scores range from 0 to 100. All adverse events and serious adverse events were recorded in accordance with National Research Ethics Service Committee guidance for trials that are not assessing an investigational medicinal product.

Quality-adjusted life-years (QALYs) were derived from participant responses to the EQ-5D-5L^17^ questionnaire, which was completed at baseline, 3 months, and 6 months. Utility values were estimated from the responses using health state utility scores based on the UK population tariff^18^, ^19^ and mapped back to the EQ-5D-3L valuation set.^20^ Costs incurred by the NHS and Personal Social Services were collected via resource utilisation questionnaires, completed at baseline and 6 months.

### Outcomes

The primary outcome was upper limb function success (defined using ARAT)^9^, ^13^ at 3 months after randomisation—ie, at the end of the intervention period. The definition of success differed depending on baseline severity: baseline ARAT score 0–7 required an improvement of 3 points or more; baseline ARAT 8–13 required an improvement of 4 points or more; baseline ARAT 14–19 required an improvement of 5 points or more; baseline ARAT 20–39 required an improvement of 6 points or more. A stepped approach was used, because although the minimal clinically important difference (MCID) for the ARAT is 10% of its range (6 points), a smaller treatment effect might still be clinically beneficial for those with severely reduced initial upper limb function, who are likely to improve less than those with more moderate reductions in function.

Secondary outcomes were upper limb function (ARAT)^9^, ^13^ success at 6 months; ARAT score; upper limb impairment (FMA);^13^, ^14^ ADL (Barthel ADL Index);^15^ quality of life (SIS, version 3.0);^16^ and upper limb pain (numerical rating scale). We also did economic analysis using data from resource utilisation questionnaires at baseline and 6 months and the EQ-5D-5L at baseline, 3 months, and 6 months.

### Statistical analysis

The target sample size was 762 participants (254 participants per group). Responses from 216 participants in each group were required to provide 80% power (significance level of 1·7% because of multiple comparisons) to detect a 15% difference in successful outcome between each of the three pairs of treatments (robot-assisted training, EULT, and usual care). The baseline estimate of success was estimated as 30% from the BoTULS trial^11^ and a difference between 30% and 45% corresponds to an odds ratio (OR) of 1·9. The sample size requirement was increased from 720 after protocol publication to allow for 15% rather than 10% attrition. Reasons for loss from the trial were recorded.

The primary outcome of upper limb function success at 3 months is reported descriptively by group and overall as a proportion. Logistic regression was used to compare success between the three groups at 3 months, adjusting for time since stroke, baseline ARAT score, and centre. We considered the possibility of partial nesting due to participants sharing therapists in the robot-assisted training and EULT groups but not in the usual care group. This did not change the model estimates or improve model fit and so the simpler models without partial nesting were used. The secondary outcomes of success at 6 months and binary coding of ARAT subscales (appendix) were analysed as for the primary outcome. All analyses were done in the intention-to-treat (ITT) population of all participants, in the group to which they were assigned, who did not have missing data after simple imputation. Preplanned sensitivity analyses were done for ARAT success at 3 months and 6 months. The first sensitivity analysis excluded participants who attended their outcome assessment outside of the 3 month ± 14 days, and 6 month ± 28 days windows. The second sensitivity analysis excluded participants with an ARAT score of zero at baseline. In a per-protocol analysis, we compared ARAT success at 3 months and 6 months for robot-assisted training versus EULT for participants who had attended at least 20 therapy sessions.

All numerical secondary outcomes were reported at baseline, 3 months, and 6 months descriptively by group as mean (SD). These secondary outcomes were analysed separately at 3 months and 6 months using linear regression adjusting for time since stroke, baseline score, and centre. The baseline score used to adjust the analysis was the same scale for that outcome—eg, baseline ARAT was used in the model for ARAT at follow-up. Bias-corrected and accelerated CIs (100 000 bootstrap intervals) have been presented for all numerical secondary outcomes because of the distribution of the data. Three preplanned subgroup analyses explored the relationship between ARAT score at 3 months and centre, time since stroke (<3 months, 3–12 months, and >12 months), and baseline ARAT scores (0, 1–7, 8–13, 14–19, and 20–39).

Simple imputation was used in the calculation of the scales.^21^ Missing values contributing to a scale or subscale total were calculated using the median value of the respondent-specific completed responses on the rest of the scale or subscale to replace missing items, if no more than 20% of items were missing. The exception was the SIS, where we used the scale developers' rules (for a particular participant, if <50% of items are missing in a dimension then the mean of the non-missing items is used in the formula for the final score; final score=25 × [mean of non-missing items–1]). For all analyses, the best fitting transformation of time since stroke was chosen on the basis of a significant reduction in model deviance. The coverage of the CIs was adjusted to account for the three paired comparisons between the groups. Because the study was powered on a significance level of 1·7%, we report 98·3% CIs. All CIs beyond the primary outcome were exploratory because no allowance was made for multiplicity of outcomes. Statistical analyses were done using Stata, version 14. Independent trial steering and data monitoring committees had oversight throughout the trial and convened annually.

A cost-utility analysis was done to assess the incremental cost per QALY gained. The analysis took the perspective of the NHS and Personal Social Services. The incremental cost per gained QALY for each participant at 6 months was calculated using the seemingly unrelated regression modelling method (sureg) in the adjusted cost-utility analysis.^22^ This method ensured that any imbalance between groups at baseline was not reflected in the estimated costs and QALYs. The covariates included in the regression analysis were centre, baseline ARAT score, time since stroke, baseline costs, and baseline utility score. To account for uncertainty in the cost-effectiveness results, a non-parametric bootstrap technique (involving 1000 replications with replacement) was used as part of a stochastic sensitivity analysis.^23^ All health economics analyses were done using Stata, version 15. The trial is registered with the ISRCTN registry, number ISRCTN69371850.

### Role of the funding source

The funder of the study had no role in study design, data collection, data analysis, data interpretation, or writing of the report. The corresponding author had full access to all the data in the study and had final responsibility for the decision to submit for publication.

## Results

**Figure 1:**
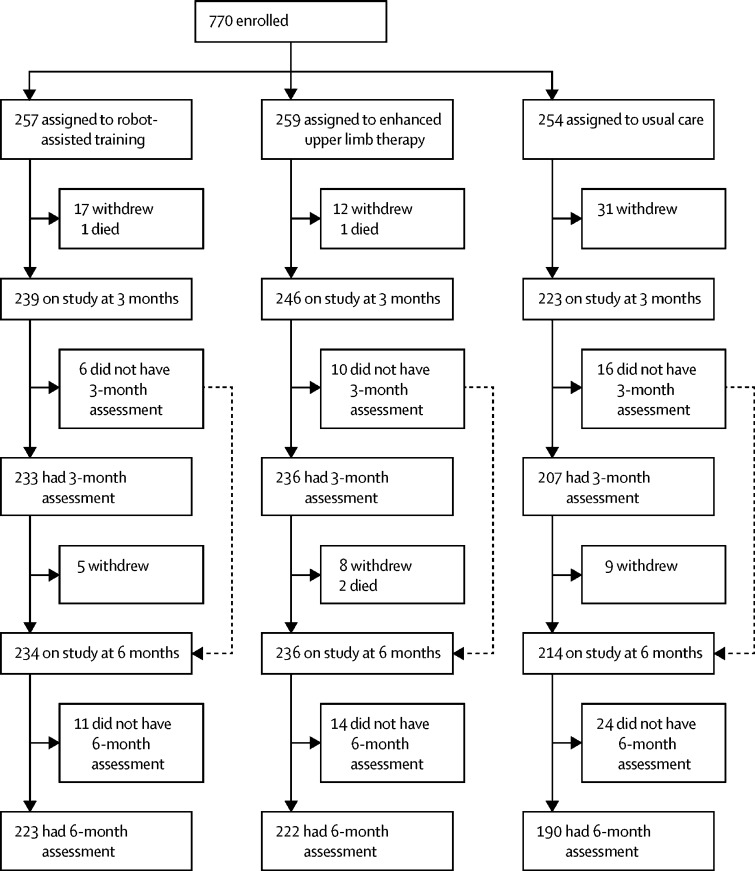
Trial profile All participants assigned to intervention groups began their trial intervention. Reasons for withdrawals and missed assessments at 3 months and 6 months are in the appendix.

Between April 14, 2014, and April 30, 2018, 770 participants were enrolled and randomly assigned to either robot-assisted training (n=257), EULT (n=259), or usual care (n=254; figure 1). Two participants who did not have a final diagnosis of stroke were withdrawn before the 3-month assessment (one in the robot-assisted training group and one in the usual care group; appendix). Of the 770 participants, 676 (88%) attended a 3-month assessment and 635 (82%) attended a 6-month assessment. Seven participants did not complete the ARAT at their 3-month assessment but were kept on study. Reasons why assessments were not done are shown in the appendix.

Baseline demographics and stroke characteristics were balanced between the groups at baseline (table 1). The mean age of participants was 61 (SD 14) years and 468 (61%) were men. The median time from stroke to randomisation was 240 days (IQR 109–549) and participants had a mean ARAT score of 8·4 (SD 11·8), mean FMA motor score of 18·1 (SD 13·7) and mean Barthel ADL Index of 14·4 (SD 3·9).

**Table 1:** Baseline demographics and clinical characteristics by randomisation group in the intention-to-treat population

		**Robot-assisted training (n=257)**	**Enhanced upper limb therapy (n=259)**	**Usual care (n=254)**
Gender				
	Female	101 (39%)	100 (39%)	101 (40%)
	Male	156 (61%)	159 (61%)	153 (60%)
Age at randomisation, years		59·9 (13·5)	59·4 (14·3)	62·5 (12·5)
Time from stroke to randomisation, days		233 (102–549)	258 (115–546)	242 (107–549)
Time from stroke to randomisation, months				
	<3	57 (22%)	46 (18%)	58 (23%)
	3–12	105 (41%)	117 (45%)	106 (42%)
	>12	95 (37%)	96 (37%)	90 (35%)
Stroke type				
	Cerebral infarction	197 (77%)	202 (78%)	214 (84%)
	Primary intracerebral haemorrhage	58 (23%)	56 (22%)	38 (15%)
	Subarachnoid haemorrhage	2 (1%)	1 (<1%)	2 (1%)
National Institute of Health Stroke Scale total score*		5·6 (3·2)	5·7 (3·2)	5·8 (3·2)
Arm affected by the stroke				
	Right	112 (44%)	116 (45%)	113 (44%)
	Left	145 (56%)	143 (55%)	141 (56%)
Handedness*				
	Right	221 (87%)	223 (86%)	228 (90%)
	Left	34 (13%)	35 (14%)	25 (10%)
	Ambidextrous	0	1 (<1%)	1 (<1%)
Action Research Arm Test†				
	Mean (SD)	8·5 (11·9)	8·7 (11·9)	8·1 (11·5)
	Median (IQR)	3·0 (0·0–11·5)	3·0 (0·0–13·0)	3·0 (0·0–11·0)
	Score 0–7	178 (70%)	175 (68%)	173 (68%)
	Score 8–13	18 (7%)	22 (8%)	23 (9%)
	Score 14–19	13 (5%)	9 (3%)	13 (5%)
	Score 20–39	47 (18%)	53 (20%)	45 (18%)
Fugl–Meyer assessment				
	Motor score*	18·0 (13·1)	18·2 (14·1)	18·2 (13·9)
	Total upper extremity score‡	68·9 (16·5)	69·0 (17·9)	68·9 (17·4)
Barthel ADL Index*		14·5 (3·8)	14·3 (4·0)	14·4 (3·9)
Numerical pain scale§		2·9 (3·2)	2·7 (3·0)	2·6 (3·1)

Data are n (%), mean (SD), or median (IQR). ADL=activities of daily living.

* Total number of patients was 255 in the robot-assisted training group.

† Total number of patients was 256 in the robot-assisted training group.

‡ Total number of patients was 254 in the robot-assisted training group.

§ Total number of patients was 253 in the robot-assisted training group.

Robot-assisted training participants attended a median of 35 (IQR 31 to 36) of the 36 sessions and EULT participants attended a median of 34 (IQR 29 to 36) of the 36 sessions. The median duration of face-to-face therapy for each attended session was 41 min (IQR 35 to 47) for robot-assisted training and 45 min (IQR 45 to 45) for EULT. The median total duration of therapy per participant over the 12-week intervention was 23 h 28 min (IQR 18 h 53 min to 25 h 46 min) for robot-assisted training and 24 h 40 min (IQR 20 h 24 min to 26 h 15 min) for EULT. The appendix shows intervention fidelity.

**Figure 2:**
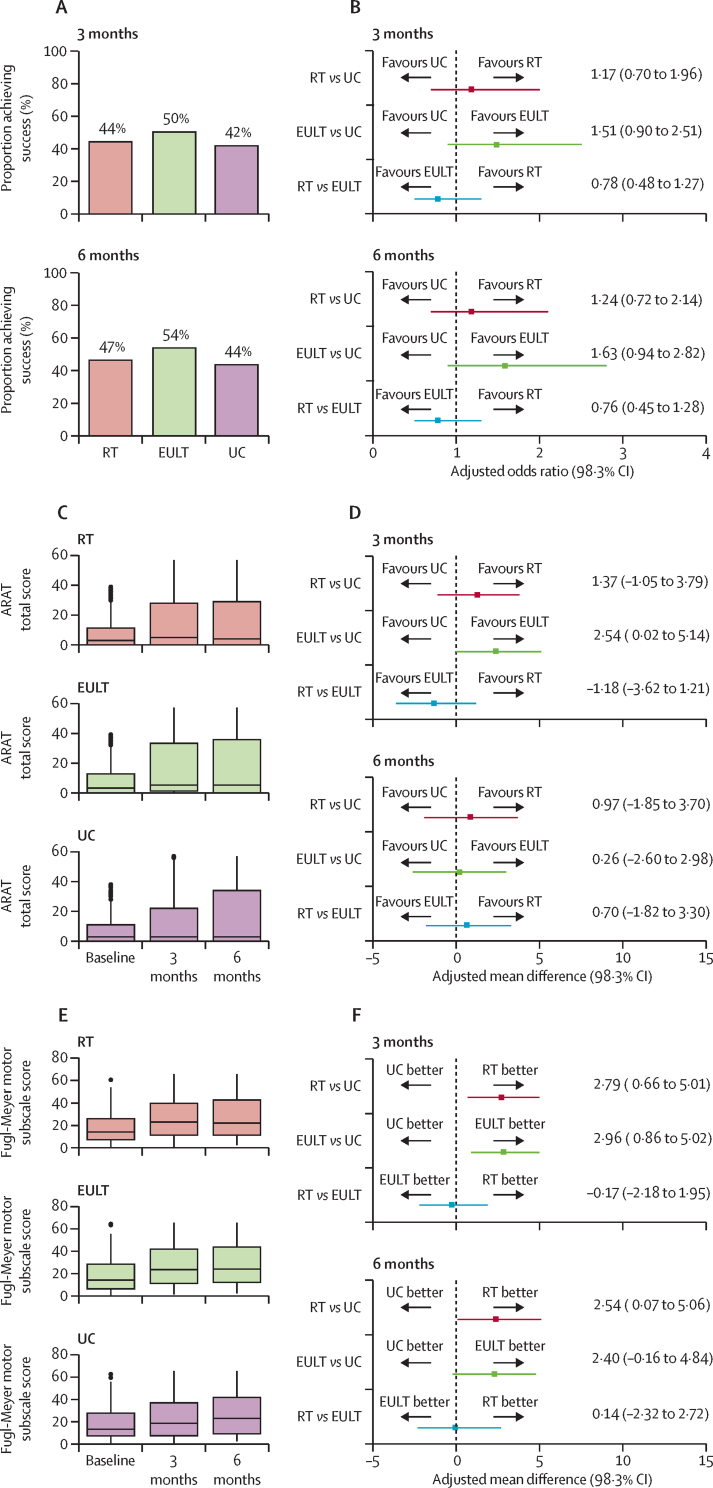
ARAT success, total ARAT score, and Fugl–Meyer motor score at baseline, 3 months, and 6 months (A) Proportion of patients achieving ARAT success. (B) Pair-wise comparison of group success. (C) ARAT total score. (D) Pair-wise comparison of ARAT total score. (E) Fugl–Meyer motor score. (F) Pair-wise comparison of Fugl–Meyer motor score. In (C) and (E), the horizontal black line is the median, the box is the IQR, and whiskers extend to the closest value within the upper or lower quartile ± 1·5 multiplied by the IQR; the black dots are any values outside of this range. ARAT=Action Research Arm Test. EULT=enhanced upper limb therapy. RT=robot-assisted training. UC=usual care.

669 participants were included in the primary outcome analysis (232 in the robot-assisted training group; 234 in the EULT group; and 203 in the usual care group). At 3 months, 103 (44%) of 232 patients in the robot-assisted training group, 118 (50%) of 234 in the EULT group, and 85 (42%) of 203 in the usual care group achieved upper limb functional recovery success (table 2; figure 2A). ARAT success was not significantly different for robot-assisted training versus EULT (adjusted odds ratio [aOR] 0·78 [98·3% CI 0·48–1·27]) or usual care (aOR 1·17 [0·70–1·96]) at 3 months (figure 2B). Similarly, comparison of EULT versus usual care showed no significant difference (aOR 1·51 [0·90–2·51]). At 6 months, 103 (47%) of 221 in the robot-assisted training group, 118 (54%) of 218 in the EULT group, and 81 (44%) of 185 in the usual care group achieved upper limb functional recovery success. Per-protocol and sensitivity analyses were consistent with the ITT analysis (table 2).

**Table 2:** Comparison of ARAT success between groups

	**Robot-assisted training**	**Enhanced upper limb therapy**	**Usual care**	**Robot-assisted training *vs* usual care**		**Enhanced upper limb therapy *vs* usual care**		**Robot-assisted training *vs* enhanced upper limb therapy**	
				Unadjusted odds ratio (98·3% CI)	Adjusted* odds ratio (98·3% CI)	Unadjusted odds ratio (98·3% CI)	Adjusted* odds ratio (98·3% CI)	Unadjusted odds ratio (98·3% CI)	Adjusted* odds ratio (98·3% CI)
**Primary outcome**†**(3 months)**									
ITT	103/232 (44%)	118/234 (50%)	85/203 (42%)	1·11 (0·70–1·76)	1·17 (0·70–1·96)‡	1·41 (0·89–2·24)	1·51 (0·90–2·51)†	0·78 (0·50–1·22)	0·78 (0·48–1·27)†
Sensitivity analysis 1	89/197 (45%)	94/187 (50%)	66/157 (42%)	1·14 (0·68–1·91)	1·11 (0·63–1·95)	1·39 (0·83–2·35)	1·39 (0·79–2·44)	0·82 (0·50–1·33)	0·80 (0·47–1·37)
Sensitivity analysis 2	71/148 (48%)	81/145 (56%)	56/116 (48%)	0·99 (0·55–1·79)	1·12 (0·56–2·22)	1·36 (0·75–2·47)	1·56 (0·78–3·12)	0·73 (0·42–1·28)	0·72 (0·38–1·38)
Per-protocol analysis	97/222 (44%)	112/219 (51%)	NA	NA	NA	NA	NA	0·74 (0·47–1·17)	0·73 (0·43–1·22)
**Secondary outcome**†**(6 months)**									
ITT	103/221 (47%)	118/218 (54%)	81/185 (44%)	1·12 (0·69–1·81)	1·24 (0·72–2·14)	1·52 (0·94–2·45)	1·63 (0·94–2·82)	0·74 (0·47–1·17)	0·76 (0·45–1·28)
Sensitivity analysis 1	91/200 (46%)	110/203 (54%)	71/163 (44%)	1·08 (0·65–1·80)	1·26 (0·71–2·23)	1·53 (0·92–2·54)	1·64 (0·93–2·90)	0·71 (0·44–1·14)	0·77 (0·45–1·31)
Sensitivity analysis 2	72/139 (52%)	84/136 (62%)	55/109 (50%)	1·06 (0·57–1·95)	1·31 (0·62–2·76)	1·59 (0·85–2·96)	1·92 (0·90–4·11)	0·67 (0·37–1·20)	0·68 (0·34–1·38)
Per-protocol analysis	97/213 (46%)	113/206 (55%)	NA	NA	NA	NA	NA	0·69 (0·43–1·10)	0·70 (0·41–1·20)

Data are n/N (%) unless otherwise stated. ARAT=Action Research Arm Test. ITT=intention to treat. NA=not applicable because a formal comparison to usual care would be biased. Per-protocol analysis=participants included if they attended at least 20 sessions of therapy. Sensitivity analysis 1=ITT patients who were assessed within the specified window of 3 months ± 14 days for 3-month assessment or 6 months ± 28 days for the 6-month assessment. Sensitivity analysis 2=ITT patients excluding those with a baseline ARAT score of zero.

* Adjusted for the variables of time since stroke, baseline ARAT score, and centre.

† Simple imputation was used in the calculation of the ARAT total score.

‡ Primary outcome analysis.

ARAT scores were similar between the robot-assisted training and usual care groups at 3 months and 6 months (figure 2C, D). Robot-assisted training participants had less upper limb impairment on the FMA motor subscale than usual care participants at 3 months (adjusted mean difference 2·79 [98·3% CI 0·66–5·01]) and the difference was sustained at 6 months (adjusted mean difference 2·54 [0·07–5·06]; table 3; figure 2E, F; MCID 4 points for acute stroke patients^24^ and 5·25 points for chronic stroke patients^25^). All other differences between robot-assisted training and usual care were non-significant. The analysis of the ARAT subscales and the descriptive statistics for the remaining secondary outcomes (FMA [range of motion and sensory subscales] and SIS [strength, emotion, memory, communication, and stroke recovery]) are in the appendix.

**Table 3:** Secondary outcomes in the intention-to-treat population

	**Robot-assisted training**		**Enhanced upper limb therapy**		**Usual care**		**Robot-assisted training *vs* usual care**		**Enhanced upper limb therapy *vs* usual care**		**Robot-assisted training *vs* enhanced upper limb therapy**	
	n	Mean (SD)	n	Mean (SD)	n	Mean (SD)	Unadjusted mean difference (98·3% CI)	Adjusted* mean difference (98·3% CI)	Unadjusted mean difference (98·3% CI)	Adjusted* mean difference (98·3% CI)	Unadjusted mean difference (98·3% CI)	Adjusted* mean difference (98·3% CI)
**ARAT score**†												
3 months	232	15·5 (19·1)	234	17·3 (20·1)	203	14·2 (19·6)	1·24 (−3·30 to 5·69)	1·37 (−1·05 to 3·79)	3·03 (−1·59 to 7·54)	2·54 (0·02 to 5·14)	−1·80 (−6·15 to 2·58)	−1·18 (−3·62 to 1·21)
6 months	221	16·5 (19·7)	218	17·2 (19·9)	185	16·4 (21·3)	0·14 (−4·80 to 5·01)	0·97 (−1·85 to 3·70)	0·78 (−4·27 to 5·64)	0·26 (−2·60 to 2·98)	−0·64 (−5·15 to 3·91)	0·70 (−1·82 to 3·30)
**FMA total score**†												
3 months	232	76·6 (22·1)	234	77·8 (22·8)	202	74·2 (23·6)	2·37 (−2·90 to 7·64)	3·12 (−0·03 to 6·50)	3·62 (−1·76 to 8·93)	3·66 (0·52 to 6·84)	−1·25 (−6·16 to 3·71)	−0·54 (−3·41 to 2·63)
6 months	221	78·2 (22·8)	218	79·4 (24·1)	186	77·9 (23·2)	0·33 (−5·20 to 5·77)	1·62 (−1·84 to 5·17)	1·46 (−4·27 to 7·03)	1·84 (−1·77 to 5·29)	−1·13 (−6·46 to 4·24)	−0·22 (−3·63 to 3·42)
**FMA: motor subscale**†												
3 months	232	26·2 (17·7)	234	27·1 (18·3)	202	24·2 (18·4)	2·03 (−2·16 to 6·17)	2·79 (0·66 to 5·01)	2·91 (−1·34 to 7·10)	2·96 (0·86 to 5·02)	−0·87 (−4·84 to 3·13)	−0·17 (−2·18 to 1·95)
6 months	221	27·2 (18·4)	217	28·2 (19·3)	186	26·3 (18·9)	0·90 (−3·59 to 5·33)	2·54 (0·07 to 5·06)	1·90 (−2·69 to 6·40)	2·40 (−0·16 to 4·84)	−0·99 (−5·32 to 3·29)	0·14 (−2·32 to 2·72)
**Barthel ADL Index**†												
3 months	233	15·5 (3·4)	236	15·9 (3·4)	207	15·3 (3·8)	0·18 (−0·65 to 1·00)	0·18 (−0·37 to 0·75)	0·65 (−0·17 to 1·47)	0·70 (0·16 to 1·25)	−0·47 (−1·22 to 0·28)	−0·51 (−1·02 to −0·01)
6 months	223	15·6 (3·4)	222	16·0 (3·5)	190	15·3 (3·7)	0·28 (−0·55 to 1·12)	0·45 (−0·12 to 1·05)	0·64 (−0·21 to 1·50)	0·88 (0·29 to 1·48)	−0·36 (−1·15 to 0·42)	−0·43 (−0·98 to 0·13)
**SIS: hand function**†												
3 months	219	15·5 (24·4)	223	21·4 (27·9)	193	14·3 (22·9)	1·26 (−4·25 to 6·83)	2·56 (−2·62 to 7·93)	7·16 (1·11 to 13·08)	7·93 (2·24 to 13·51)	−5·90 (−11·85 to 0·12)	−5·37 (−10·86 to 0·18)
6 months	213	15·7 (25·2)	216	18·4 (26·2)	179	14·8 (23·5)	0·94 (−4·99 to 6·78)	1·94 (−3·56 to 7·47)	3·60 (−2·42 to 9·52)	3·91 (−1·76 to 9·59)	−2·65 (−8·58 to 3·35)	−1·96 (−7·40 to 3·59)
**SIS: mobility**†												
3 months	219	61·6 (25·1)	223	66·4 (23·2)	192	61·0 (24·6)	0·64 (−5·27 to 6·45)	1·31 (−4·30 to 6·87)	5·44 (−0·13 to 11·11)	5·81 (0·45 to 11·20)	−4·80 (−10·35 to 0·71)	−4·50 (−9·87 to 0·88)
6 months	213	61·7 (24·8)	216	63·9 (23·7)	180	62·9 (23·8)	−1·19 (−7·05 to 4·73)	−0·46 (−6·00 to 5·09)	1·07 (−4·65 to 6·79)	1·44 (−4·06 to 6·83)	−2·26 (−7·91 to 3·32)	−1·90 (−7·28 to 3·48)
**SIS: ADL**†												
3 months	220	50·8 (22·5)	223	55·9 (19·8)	194	50·8 (21·1)	0·004 (−5·11 to 5·20)	0·75 (−4·16 to 5·77)	5·13 (0·30 to 9·91)	5·55 (0·87 to 10·21)	−5·13 (−9·97 to −0·27)	−4·81 (−9·48 to −0·12)
6 months	212	50·4 (22·3)	216	52·5 (22·3)	179	51·9 (21·3)	−1·42 (−6·68 to 3·88)	−0·56 (−5·60 to 4·52)	0·59 (−4·65 to 5·85)	1·10 (−3·90 to 6·13)	−2·01 (−7·18 to 3·14)	−1·66 (−6·66 to 3·31)
**SIS: social participation**†												
3 months	217	47·7 (24·7)	221	51·7 (23·0)	193	47·1 (23·7)	0·62 (−5·14 to 6·32)	0·54 (−5·18 to 6·19)	4·64 (−0·90 to 10·11)	4·70 (−0·75 to 10·13)	−4·02 (−9·48 to 1·47)	−4·16 (−9·57 to 1·34)
6 months	210	47·0 (25·9)	216	50·2 (24·4)	179	49·2 (23·8)	−2·24 (−8·30 to 3·87)	−2·21 (−8·23 to 3·87)	1·02 (−4·84 to 6·86)	1·22 (−4·66 to 7·03)	−3·26 (−9·07 to 2·62)	−3·43 (−9·16 to 2·43)
**Upper limb pain (numerical rating scale)**												
3 months	232	2·5 (3·2)	236	2·2 (2·9)	206	2·7 (3·2)	−0·19 (−0·93 to 0·54)	−0·24 (−0·90 to 0·42)	−0·55 (−1·26 to 0·15)	−0·55 (−1·20 to 0·09)	0·36 (−0·32 to 1·03)	0·31 (−0·31 to 0·93)
6 months	223	2·5 (3·2)	221	2·5 (3·2)	190	2·0 (2·9)	0·52 (−0·22 to 1·24)	0·37 (−0·32 to 1·06)	0·48 (−0·26 to 1·20)	0·36 (−0·34 to 1·06)	0·04 (−0·69 to 0·77)	0·01 (−0·68 to 0·70)

Data are n or mean (SD). ARAT=Action Research Arm Test. FMA=Fugl–Meyer Assessment. ADL=activities of daily living. SIS=Stroke Impact Scale.

* Adjusted for the variables of time since stroke, baseline scale score (not available for SIS), and centre.

† Simple imputation was used in the calculation of the score.

Participants who had robot-assisted training performed less well in the ADL tests at 3 months than those allocated to EULT (table 2): Barthel ADL Index adjusted mean difference −0·51 (98·3% −1·02 to −0·01; MCID 1·85)^25^ and SIS ADL adjusted mean difference −4·81 (−9·48 to −0·12; MCID 5·9).^25^ Other measures were not different between the robot-assisted training and EULT groups.

Compared with participants who received usual care, EULT patients had less upper limb impairment on the FMA total score at 3 months (adjusted mean difference 3·66 [98·3% CI 0·52–6·84]) and FMA motor subscale score (adjusted mean difference 2·96 [0·86–5·02]; table 3; MCID 4 for patients with acute stroke and 5·25 for those with chronic stroke). Participants who received EULT had better upper limb function than those who had usual care in terms of ARAT total score (adjusted mean difference 2·54 [0·02–5·14]; figure 2D; table 3; MCID 6) at 3 months. Participants who had EULT scored higher on the Barthel ADL Index than those who had usual care (adjusted mean difference 0·70 [0·16–1·25] at 3 months and 0·88 [0·29–1·48] at 6 months; MCID 1·85). Compared with usual care, patients who had EULT performed better on SIS hand function (7·93 [2·24–13·51]; MCID 17·8), mobility (5·81 [0·45–11·20]; MCID 4·5), and ADL (5·55 [0·87–10·21]; MCID 5·9) at 3 months. No significant differences were recorded in the pain numerical rating scale tests.

The outcome assessors reported that they were unmasked for 50 (21%) of 233 participants at 3 months and 39 (17%) of 223 at 6 months in the robot-assisted training group, 26 (11%) of 236 participants at 3 months and 25 (11%) of 220 at 6 months in the EULT group, and 25 (12%) of 207 at 3 months and 24 (13%) of 190 at 6 months in the usual care group.

43 serious adverse events were reported for 39 participants in the robot-assisted training group, 42 were reported for 33 participants in the EULT group, and 29 were reported for 20 participants in the usual care group (appendix). None of the serious adverse events were related to a trial intervention. The median number of serious adverse events was zero (IQR 0–0) across all three groups. More serious adverse events occurred in the robot-assisted training group than in the usual care group (Mann-Whitney U test p=0·013), but other groups had similar numbers (robot-assisted training *vs* EULT p=0·45; EULT *vs* usual care p=0·08). These differences are probably due to a reporting bias, because those in the intervention groups had regular contact with clinical teams—eg, six serious adverse events were reported for robot-assisted training participants by the local principal investigator, which did not result in hospitalisation or death.

There was no significant difference between the groups for mean ARAT score at 3 months within the prespecified subgroups of centre, time since stroke, or baseline ARAT score, although the 98·3% CIs were wide as expected because of the reduced sample size of the subgroups (appendix).

The unadjusted results of the economic analysis suggest that, on average, usual care was the least costly option at 6 months (£3785 per participant) with robot-assisted training being the most costly (£5387 per participant; table 4). The average cost of EULT per participant was £4451; however, EULT had higher QALYs (0·229) than usual care (0·212) or robot-assisted training (0·212) at 6 months. The incremental cost per QALY at 6 months for participants in the EULT group compared with those in the usual care group was £74 100, with a 19% chance of being cost-effective at the £20 000 willingness to pay threshold. Throughout the analysis, results suggested that robot-assisted training was more costly than usual care and EULT, and was no more effective than EULT or usual care.

**Table 4:** Results from base-case and probabilistic cost-utility analysis for usual care, EULT, and robot-assisted training

	**Unadjusted mean cost (98·3% CI)**	**Unadjusted mean QALYs (98·3% CI)**	Adjusted*incremental QALY (98·3% CI)	Adjusted*incremental costs (98·3% CI)	**Adjusted incremental cost-effectiveness ratio**	**Probability that each therapy is cost effective at different willingness to pay thresholds**†				
						£0	£10 000	£20 000	£30 000	£50 000
Robot-assisted training	£5387 (4777 to 5996)	0·212 (0·195 to 0·229)	..	..	More expensive and less effective than EULT in both adjusted and unadjusted analyses	0%	0%	0%	0%	0%
Enhanced upper limb therapy	£4451 (3548 to 5354)	0·229 (0·213 to 0·244)	0·010 (−0·005 to 0·025)	741 (−461 to 1943)	£74 100	10%	15%	19%	26%	38%
Usual care	£3785 (2801 to 4770)	0·212 (0·194 to 0·230)	..	..	..	90%	85%	81%	74%	62%

Numbers of patients included in analyses were 178 in the usual care group, 259 in the EULT group, and 257 in the robot-assisted training group for the unadjusted cost calculation; 254 in the usual care group, 259 in the EULT group, and 257 in the robot-assisted training group for the unadjusted QALY calculation; and 171 in the usual care group, 254 in the EULT group, and 247 in the robot-assisted training group for the adjusted analyses. EULT=enhanced upper limb therapy. QALY=quality-adjusted life-year.

* Adjusted analysis done using the seemingly unrelated regression (sureg) function on STATA, version 15; adjusted for centre, baseline ARAT score, time since stroke, baseline costs, and baseline utility score; performed for the comparison between usual care and EULT as the next best alternative.

† The probabilistic sensitivity analysis includes all three therapies and was done for different threshold values of society's willingness to pay per QALY (£0, £10 000, £20 000, £30 000, and £50 000).

## Discussion

We found no difference in upper limb function, defined as ARAT success, between stroke patients treated with robot-assisted training using the MIT-Manus robotic gym, a EULT programme, or usual care. All groups improved on this measure from baseline to 3 months, which was maintained at 6 months. Some differences in secondary outcomes suggest potential benefits for EULT and robot-assisted training that might have implications for clinical practice and future research. We found no safety concerns for either intervention. Neither robot-assisted training nor EULT, when delivered with a one-to-one patient-to-therapist ratio, would be considered cost-effective at the UK current level of willingness to pay per QALY (£20 000–30 000).

Of the many preplanned comparisons of the secondary outcomes, some indicated differences that are likely to be clinically important, because the MCID is within the 98·3% CI. Robot-assisted training improved upper limb impairment as measured by the FMA motor subscale at 3 months compared with usual care and this difference was maintained at 6 months. However, this improvement did not translate into improvement in either upper limb function or ADL. Indeed, robot-assisted training participants did less well in ADL at 3 months than those who received EULT. EULT led to improvements in upper limb impairment, mobility, and ADL compared with usual care at 3 months. No clinically important differences were found between EULT and usual care or robot-assisted training and EULT at 6 months on any outcome measure.

Although there was no significant difference between EULT and usual care in ARAT success, the absolute difference was 8% at 3 months and 10% at 6 months in favour of EULT. This difference might be considered important by some patients and clinicians, but the trial did not have the statistical power to detect a difference of this size.

The RATULS trial has a number of strengths and weaknesses. The risk of bias was low. As the study progressed, we abandoned the screening log because this became a disincentive for clinical teams to refer potential participants. Randomisation was by an independent service with allocation concealment. The outcome measures are widely used in stroke rehabilitation trials and have been shown to be valid, reliable, and sensitive to change.^26^ However, the primary outcome, derived from the ARAT, was developed specifically for the RATULS trial and further validation is needed. All outcomes were analysed according to statistical and health economic analysis plans. There were low levels of missing data for all assessments. Overall attrition rates were acceptable but were higher in the usual care group (20%) than in the robot-assisted training and EULT groups (both 10%), and differential attrition is a potential source of bias. Most of the withdrawals before 3 months in usual care were due to disappointment with treatment allocation.

There was robust development of robot-assisted training and EULT programmes, and efforts to promote fidelity. Robot-assisted training was compared to an upper limb therapy programme of the same frequency and duration based on best evidence and best practice. Experts in both robot-assisted training and EULT were involved in designing and delivering the training programmes. Both interventions have been described according to the Template for Intervention Description and Replication, enabling them to be replicated in further research or clinical practice.^8^ Robot-assisted training was provided as it would be in clinical practice, while EULT was provided as a centralised rather than a local service for logistical reasons. Intervention delivery was monitored throughout the trial with high levels of compliance.

RATULS was a pragmatic trial and therefore usual care was chosen as a comparator. Obtaining accurate information about the usual care that patients receive is a challenge for this type of study and the assumption that usual care is uniformly provided is rarely true. Although UK guidelines suggest the amount of therapy that a stroke patient should receive, they refer to contact time with a therapist rather than specifying the focus or intensity.^27^ Disappointment about group allocation might have resulted in some usual care participants seeking or being provided with additional therapy, or increasing the amount of self-practice exercises they did, thereby introducing a competitive therapy bias. We did consider offering either robot-assisted training or EULT to usual care participants after the 6-month outcome assessment, but this was not feasible.

The 2018 Cochrane systematic review of electromechanical and robot-assisted arm training (45 trials, 1619 participants) reported significantly improved ADL scores (standardised mean difference [SMD] 0·31 [95% CI 0·09–0·50] and arm function (SMD 0·32 [95% CI 0·18–0·46]) at the end of the intervention period.^5^ Although this review described improvement in arm function, the FMA motor subscale, a measure of upper limb impairment, was reported as the most commonly used arm function outcome measure.

The robot-assisted training programme was provided at the same frequency and duration as intended in the VA Robotics Trial,^28^ which also assessed the MIT-Manus robotic gym. The VA Robotics Trial also found that robot-assisted training improved upper limb impairment (FMA motor subscale) compared with usual care, but the impairment advantage did translate into significant upper limb functional improvements (Wolf Motor Function Test) and benefit in the SIS.^28^ The intensive comparative therapy in the VA Robotics trial sought to replicate the form and intensity of upper limb movements provided by MIT-Manus, and there were no significant differences between this and the robot-assisted training group. A strength of the RATULS trial is that robot-assisted training was compared to an upper limb therapy programme, based on best evidence and best practice, of the same frequency and duration. The VA Robotics Trial reported that the average total cost of treatment over the intervention period was US$5152 for robot-assisted training and $7382 for intensive comparison therapy (p=0·001). In the VA Robotics Trial, the base-case analysis assumed that the MIT-Manus robotic gym was used by two patients simultaneously. At 36 weeks, the total health-care costs were similar for all three groups.^29^ The REM-AVC trial^30^ assigned participants to receive robot-assisted training with an Armeo Spring device or self-rehabilitation of the same frequency and duration, and found no difference between groups for the FMA.

It is important to consider why the improvements in impairment seen with robot-assisted training in the RATULS trial did not translate into improved function. The RATULS trial provided integrated training with all three modules of the MIT-Manus robotic gym (shoulder–elbow module; wrist module; and hand module), which adapts to participants' abilities (providing more or less assistance as required). The robot-assisted training programme did not include grip or pinch activities. Participants trained specific movements of their affected arm in a spatially controlled manner, but it is possible that these were not considered meaningful by participants and there might have been insufficient guidance for participants about making the best use of any reduction in impairment in day-to-day activities. Many daily activities involve both upper limbs in bilateral (eg, opening a drawer) or bimanual (eg, making a sandwich) coordinated action. This could explain why robot-assisted training resulted in less favourable outcomes in self-reported ADL compared with EULT, in which training specifically focused on daily activities and functional tasks. The improvement in mobility seen after EULT would also support this theory, because EULT included tasks involving the upper limbs in balance and sit-to-stand activities, which were not components of robot-assisted training.

The EULT programme was based on goal-orientated repetitive functional task practice, which resulted in some potentially clinically important benefits over usual care and improvements in upper limb impairments that were translated into improvement in ADL. This supports the conclusions of the 2016 Cochrane review (33 trials, 1853 participants), which found that repetitive functional task practice improved arm function (SMD 0·25 [95% CI 0·01–0·49]), ADL (SMD 0·28, [0·10–0·45]), and hand function (SMD 0·25 [0·00–0·51]).^3^ The review did not include the Graded Repetitive Arm Supplementary Program (GRASP) trial,^31^ which found significant improvements in upper limb function, grip strength, and upper limb use in daily activities. Participants in the RATULS trial had more severe upper limb impairment than those in the GRASP trial. Delivering EULT as group or classroom therapy would lead to a reduction in costs. However, the impact of this change on QALYs is unknown and should be explored.

It is likely that the pragmatic inclusion criteria led to the recruitment of some participants who had little prospect of recovery. Approaches that stratify patients into groups with differing probabilities of upper limb recovery, such as advanced neuroimaging and transcranial magnetic stimulation, have been developed and should be considered in future trials to improve the targeting of therapies towards participants with the most potential to respond.^32^ Ongoing biomarker research might help in patient selection or treatment monitoring of future stroke rehabilitation trials.^33^ Further research is needed to see how improvements in upper limb impairment, seen with robot-assisted training, can translate into functional gain.

We recruited patients with acute, subacute, and chronic stroke in our trial because there was no evidence to exclude patients at any stage of recovery. The Cochrane systematic review on repetitive task training also found no influence of time since stroke on functional outcomes.^3^ Subgroup analyses of the RATULS trial showed no clear effect of time since stroke on the effectiveness of the interventions.

There is converging evidence that more therapy might result in better outcomes,^34^ but in future, adequately powered dose-finding studies of promising interventions, tailored to targeted subgroups, are needed, which also take into account potential cost-effectiveness. Future trials might wish to consider having a co-primary outcome measure of a patient-reported outcome measure—eg, SIS—as well as one of the standard measures of impairment and functional limitation.

In summary, the RATULS trial did not find evidence that a robot-assisted training programme using the MIT-Manus robotic gym improved upper limb function (measured by ARAT success) after a stroke when compared with an EULT programme of the same frequency and duration, or usual care. Robot-assisted training led to improvement in upper limb impairment (FMA motor subscale) compared with usual care but not improvements in upper limb function or ADL. EULT led to improvements in upper limb impairment (FMA motor subscale), mobility (SIS), and ADL (SIS) compared with usual care at the end of the intervention period (at 3 months). Neither robot-assisted training nor EULT was cost-effective.

## Data sharing

The trial protocol is published.^8^ De-identified participant data will be made available to scientific researchers on approval of their study protocol and analysis plan by a committee of the RATULS team. Proposals should be directed to the corresponding author. A data sharing agreement will need to be signed by data requestors. Requests for data sharing will be considered after publication of the full trial report in the Health Technology Assessment Journal, which is anticipated in 2020.
