# Supplementary material for: Robot assisted training for the upper limb after stroke (RATULS): a multicentre randomised controlled trial
Source: Lancet. 2019 Jul 6;394(10192):51–62. doi: 10.1016/S0140-6736(19)31055-4 (PMC6620612; doi:10.1016/S0140-6736(19)31055-4)
Supplement: Supplementary appendix [file mmc1.pdf]

THE LANCET

Supplementary appendix

This appendix formed part of the original submission and has been peer reviewed. We post it as supplied by the authors.

Supplement to: Rodgers H, Bosomworth H, Krebs HI, et al. Robot assisted training for the upper limb after stroke (RATULS): a multicentre randomised controlled trial. *Lancet* 2019; published online May 22. http://dx.doi.org/10.1016/S0140-6736(19)31055-4.

Supplementary Table 1: **Reasons for withdrawal or assessment not conducted at three months**

**Reasons for withdrawal/ assessment not conducted at 3 months**	**RT**	**EULT**	**UC**	**Total**
**Assessment not conducted**	**6**	**10**	**16**	**32**
Participant did not attend agreed scheduled appointment and did not respond to attempts to rearrange	2	2	3	7
Participant did not respond to phone/email/postal attempts to arrange assessment	2	5	7	14
Participant had a change in personal circumstances and was unable to attend assessment	1	0	1	2
Participant refused to complete assessment – reason unknown	0	1	0	1
Participant unwell and unable to do assessment	1	2	5	8
**Died**	**1**	**1**	**0**	**2**
Died	1	1	0	2
**Withdrawn**	**17**	**12**	**31**	**60**
Participant did not wish to take part as randomised to usual care	0	0	20	20
Participant experienced pain during therapy sessions	0	1	0	1
Participant experienced upper limb pain	1	1	0	2
Participant found to be ineligible, diagnosis not stroke	1	0	1	2
Participant had a change in personal circumstances and was unable to continue participation in the study	2	0	0	2
Participant moved out of study area	1	1	0	2
Participant refused to complete assessment – reason unknown	0	1	0	1
Participant refused to continue participation in the study as they did not feel any improvement in arm	1	0	0	1
Participant refused to continue participation in the study as they felt it was too much burden	4	6	9	19
Participant unwell and unable to continue participation in the study	7	2	1	10
**Total**	**24**	**23**	**47**	**94**

Supplementary Table 2: **Reasons for withdrawal or assessment not conducted at six months**

**Reasons for withdrawal/ assessment not conducted at 6 months**	**RT**	**EULT**	**UC**	**Total**
**Assessment not conducted**	**11**	**14**	**24**	**49**
Participant did not attend agreed scheduled appointment and did not respond to attempts to rearrange	2	1	2	5
Participant did not respond to phone/email/postal attempts to arrange assessment	6	12	19	37
Participant had a change in personal circumstances and was unable to attend assessment	1	0	0	1
Participant paperwork was lost	1	0	0	1
Participant refused to complete assessment - reason unknown	0	0	1	1
Participant unwell and unable to do assessment	1	1	2	4
**Died**	**0**	**2**	**0**	**2**
Died	0	2	0	2
**Died before 3 month assessment**	**1**	**1**	**0**	**2**
**Withdrawn**	**5**	**8**	**9**	**22**
Participant behaviour was inappropriate	0	1	0	1
Participant had a change in personal circumstances and was unable to continue participation in the study	0	0	1	1
Participant had problems with transport arrangements to assessment	0	0	1	1
Participant moved out of study area	0	1	0	1
Participant refused to continue participation in the study (reason unknown)	2	3	1	6
Participant refused to continue participation in the study as they did not feel any improvement in arm	0	1	0	1
Participant refused to continue participation in the study as they felt it was too much burden	0	1	3	4
Participant unhappy with randomisation group	0	0	1	1
Participant unwell and unable to continue participation in the study	3	1	2	6
**Withdrawn before 3 month assessment**	**17**	**12**	**31**	**60**
**Total**	**34**	**37**	**64**	**135**

Supplementary Table 3: **Session attendance, duration of therapy received by RT and EULT participants.**

Study centre (number of participants)		Number of sessions attended out of the expected 36 sessions per participant	Duration of therapy within each attended therapy/training session (minutes)	Total duration of therapy for therapy/training programmes (hr:minutes)	Reasons for missed therapy/ training sessions (pt=number of participant, n=number of missed sessions)
RT Total (n=257)	*Number of sessions attended/ expected (%)*	8026/9252 (87%)	n/a	n/a	Did not attend (reason unknown) (pt=23, n=132)<br>Fatigue (pt=3, n=15)<br>Participant being discharged from hospital at time of therapy (pt=1, n=1)<br>Participant cancelled session (family emergency) (pt=2, n=2)<br>Participant could not tolerate three sessions per week (pt=3, n=31)<br>Participant could not tolerate the wrist robot (pt=3, n=41)<br>Participant died (pt=1, n=32)<br>Participant forgot appointment (pt=2, n=2)<br>Participant had another appointment (pt=29, n=69)<br>Participant moved area (pt=1, n=8)<br>Participant on holiday (pt=6, n=15)<br>Participant unwell (pt=52, n=341)<br>Participant unable to leave house due to access problems (pt=1, n=1)<br>Personal circumstances (pt=5, n=54)<br>Problems with transport to appointment (pt=14, n=22)<br>Robot out of order (pt=1, n=1)<br>Shoulder pain (pt=5, n=62)<br>Therapy slot unavailable (e.g. bank holiday) (pt=7, n=14)<br>Withdrew (pt=15, n=429)
	Min	0	1	0 hr 0 min	
	Max	40	90	33 hr 8 min	
	Median	35 [31-36]	41 [35-47]	23 hr 28 min [18 hr 53 min -25 hr 46 min]	
	n	257	255	257	
EULT Total (n=259)	*Number of sessions attended/ expected (%)*	7877/9324 (84 %)	n/a	n/a	Adverse weather conditions (pt=3, n=5)<br>Did not attend (reason unknown) (pt=23, n=96)<br>Fatigue (pt=5, n=14)<br>Participant at work (pt=3, n=4)<br>Participant attended one therapy session per week (pt=1, n=23)<br>Participant being discharged from hospital at time of therapy (pt= 6, n=9)<br>Participant cancelled session (reason unknown) (pt=7, n= 27)<br>Participant cancelled session (family emergency) (pt=2, n=5)<br>Participant forgot appointment (pt=9, n=10)<br>Participant had another appointment (pt=54, n=115)<br>Participant moved area (pt=1, n=35)<br>Participant on holiday (pt=21, n=117)<br>Participant overslept (pt=2, n=3)<br>Participant unhappy with randomisation group (pt=1, n=31)<br>Participant unwell (pt=67, n = 406)<br>Participant unable to leave house due to access problems (pt=2, n=26)<br>Participant working away from home (pt=2, n=50)<br>Personal circumstances (pt=12, n= 88)<br>Problems with transport to appointment (pt=32, n =70)<br>Shoulder pain (pt=2, n=4)<br>Therapy slot unavailable (e.g. bank holiday) (pt=20, n=27)<br>Withdrew (pt=11, n=282)
	Min	1	0	0hr 55min	
	Max	36	90	36hr 31min	
	Median	34 [29-36]	45 [45-45]	24hr 40min [20hr 24min-26hr 15min]	
	n	259	183	183	

Data are median [IQR], or n/N (%)

Supplementary materials for Robot Assisted Training for the Upper Limb after Stroke (RATULS): a multi-centre randomised controlled trial comparing robot-assisted training; an enhanced upper limb therapy programme; and usual care.

Analysis of the ARAT subscales

The distribution of the data was "U" shaped and almost binary as participants mostly scored 0 or full marks on each subscale. Therefore we created a binary variable for each ARAT subscale which is "Some" if the participant gets a 2 or 3 for any of the items in that subscale and is "None" otherwise. The rationale is that we would then be comparing patients who can complete at least one task fully compared to those who could not.

Supplementary Table 4: **Comparison of 'some' on ARAT subscales at three and six months between trial randomisation groups.**

		RT		EULT		UC		Odds ratio (98⅓% CI) for RT vs UC(2)		Odds ratio (98⅓% CI) for EULT vs UC(2)		Odds ratio (98⅓% CI) for RT vs EULT(2)	
		n	Number (%)	n	Number (%)	n	Number (%)	Unadjusted	Adjusted(1)	Unadjusted	Adjusted(1)	Unadjusted	Adjusted(1)
Grasp(2)	Baseline	256	55 (21·5%)	259	58 (22·4%)	254	43 (16·9%)	NA					
	3 months	232	77 (33·2%)	234	83 (35·5%)	203	55 (27·1%)	1·34 (0·81,2·21)	1·55 (0·75,3·22)	1·48 (0·90,2·44)	1·63 (0·79,3·38)	0·90 (0·57,1·44)	0·95 (0·49,1·85)
	6 months	221	83 (37·6%)	218	75 (34·4%)	185	57 (30·8%)	1·35 (0·81,2·24)	1·81 (0·87,3·75)	1·18 (0·71,1·96)	1·02 (0·48,2·20)	1·15 (0·71,1·85)	1·77 (0·88,3·54)
Grip(2)	Baseline	256	61 (23·8%)	259	58 (22·4%)	254	55 (21·7%)	NA					
	3 months	232	85 (36·6%)	234	91 (38·9%)	203	62 (30·5%)	1·32 (0·81,2·14)	1·86 (0·86,4·02)	1·45 (0·89,2·35)	2·39 (1·12,5·12)	0·91 (0·58,1·44)	0·78 (0·40,1·52)
	6 months	221	79 (35·7%)	218	90 (41·3%)	185	65 (35·1%)	1·03 (0·62,1·69)	1·15 (0·54,2·45)	1·30 (0·79,2·13)	1·76 (0·85,3·66)	0·79 (0·49,1·27)	0·66 (0·33,1·30)
Pinch(2)	Baseline	256	29 (11·3%)	259	32 (12·4%)	254	30 (11·8%)	NA					
	3 months	232	54 (23·3%)	234	58 (24·8%)	203	42 (20·7%)	1·16 (0·67,2·03)	1·60 (0·78,3·28)	1·26 (0·73,2·19)	1·64 (0·80,3·34)	0·92 (0·55,1·55)	0·98 (0·50,1·89)
	6 months	221	53 (24·0%)	218	58 (26·6%)	185	49 (26·5%)	0·88 (0·51,1·52)	1·04 (0·52,2·11)	1·01 (0·59,1·73)	1·13 (0·57,2·27)	0·87 (0·51,1·47)	0·92 (0·47,1·79)
Gross m ovement(2)	Baseline	256	85 (33·2%)	259	94 (36·3%)	254	78 (30·7%)	NA					
	3 months	232	100 (43·1%)	234	110 (47·0%)	203	80 (39·4%)	1·16 (0·73,1·86)	1·35 (0·73,2·49)	1·36 (0·86,2·17)	1·40 (0·75,2·59)	0·85 (0·55,1·33)	0·96 (0·53,1·74)
	6 months	221	103 (46·6%)	218	107 (49·1%)	185	76 (41·1%)	1·25 (0·77,2·03)	1·68 (0·87,3·22)	1·38 (0·85,2·24)	1·52 (0·78,2·94)	0·91 (0·57,1·43)	1·11 (0·60,2·04)

Data are n (%), odds ratio (CI) (1) for time since stroke$^{-0.5}$, baseline ARAT and centre (2) Simple imputation was used in the calculation of the ARAT score. NA = Not applicable.
RT, robot-assisted training; EULT, enhanced upper limb therapy; UC, usual care.

Supplementary Table 5: **Descriptive statistics for subscales at three and six months by randomisation groups**

			RT		EULT		UC	
			**n**	**Mean (SD)**	**n**	**Mean (SD)**	**n**	**Mean (SD)**
**Upper limb impairment (Fugl-Meyer Assessment)**	**Range of motion and joint pain[1]**	**Baseline**	254	41·5 (6·1)	259	41·5 (6·2)	254	41·0 (6·6)
		**3 months**	232	40·8 (6·8)	234	41·2 (6·2)	204	40·0 (8·0)
		**6 months**	221	41·5 (6·1)	218	41·6 (5·9)	186	41·6 (6·2)
	**Sensation[1]**	**Baseline**	253	9·4 (3·2)	258	9·4 (3·3)	252	9·8 (2·9)
		**3 months**	231	9·6 (3·1)	234	9·5 (3·0)	202	9·9 (2·9)
		**6 months**	221	9·5 (3·0)	218	9·6 (2·9)	186	10·0 (2·7)
**Quality of life (Stroke Impact Scale)**	**Strength[1]**	**3 months**	220	43·8 (20·4)	220	48·1 (22·2)	193	40·1 (19·1)
		**6 months**	213	40·9 (21·7)	216	44·3 (22·7)	176	40·3 (21·6)
	**Emotion[1]**	**3 months**	220	68·3 (18·2)	222	69·9 (16·5)	194	67·4 (20·5)
		**6 months**	211	66·9 (18·3)	216	68·3 (18·8)	179	67·4 (19·7)
	**Memory[1]**	**3 months**	219	76·0 (22·4)	222	80·3 (18·8)	194	76·8 (22·2)
		**6 months**	213	74·6 (22·7)	215	77·4 (21·2)	178	77·8 (23·4)
	**Communication[1]**	**3 months**	220	80·1 (23·4)	222	85·3 (20·5)	194	80·9 (24·3)
		**6 months**	213	79·3 (21·7)	216	83·7 (22·7)	179	82·2 (22·5)
	**Stroke recovery[1]**	**3 months**	217	47·9 (18·9)	223	50·3 (18·9)	190	45·2 (19·9)
		**6 months**	213	51·4 (20·4)	215	50·9 (21·5)	180	48·5 (20·3)

Data are mean (SD). [1] Simple imputation was used in the calculation of the subscale.

RT, robot-assisted training; EULT, enhanced upper limb therapy; UC, usual care.

Supplementary Table 6: **Serious adverse events (SAEs) in the intention to treat population**

	RT SAEs n=43	EULT SAEs n=42	UC SAEs n=29
**Event resulted in death[1]: n(%)**	1 (2·3%)	4 (9·5%)	1 (3·4%)
**Event was life threatening: n(%)**	3 (7·0%)	0 (0·0%)	0 (0·0%)
**Event resulted in admission to hospital or prolongation of hospitalisation: n(%)**	32 (74·4%)	37 (88·1%)	25 (86·2%)
**Event resulted in persistent or significant disability or incapacity: n(%)**	1 (2·3%)	0 (0·0%)	3 (10·3%)
**Event was 'otherwise considered significant' by the investigator: n(%)**	6 (14·0%)	1 (2·4%)	0 (0·0%)

Data are n (%) [1] For two participants (EULT n=1 and UC n=1), a SAE was initially reported during their involvement in the trial but the date of death from this event was after their involvement ceased. Mann-Whitney-U test on the number of SAEs per participant for RT vs UC p=0·013, RT vs EULT p=0·40, EULT vs UC p=0·08.

Supplementary Table 7: **Summarised SAEs which resulted in death**

	RT	EULT	UC
**Neurological**	**0**	**1**	**1**
Glioblastoma multiforme [a]	0	0	1
New stroke [a]	0	1	0
**Respiratory**	**0**	**1**	**0**
Pneumonia	0	1	0
**Urinary tract**	**0**	**2**	**0**
Metastatic renal carcinoma	0	1	0
Urosepsis	0	1	0
**Miscellaneous**	**1**	**0**	**0**
Suicide	1	0	0
**Total**	**1**	**4**	**1**

[a] These events commenced during study involvement but date of death was after study end.

Supplementary Table 8: **Summarised SAEs which did not result in death**

**SAE Event**	**RT**		**EULT**		**UC**	
	**No of participants experienced event**	**Total number of events**	**No of participants experienced event**	**Total number of events**	**No of participants experienced event**	**Total number of events**
**Cardiovascular**	**5**	**5**	**2**	**2**	**0**	**0**
Aortic aneurysm rupture		1		0		0
Elective carotid endarterectomy		0		1		0
Hypotension		1		0		0
NSTEMI		1		0		0
Paroxysmal atrial fibrillation		1		0		0
Possible angina		0		1		0
Vasovagal collapse		1		0		0
**Endocrine**	**0**	**0**	**1**	**1**	**1**	**1**
Amiodarone-induced thyrotoxicosis		0		1		0
Hyperglycaemia		0		0		1
**Ear Nose and Throat**	**0**	**0**	**1**	**1**	**0**	**0**
Elective vocal cord medialisation		0		1		0
**Gastrointestinal**	**4**	**4**	**6**	**7**	**1**	**1**
Bleeding oesophageal ulcers		0		1		0
Elective endoscopy for diarrhoea		1		0		0
Elective hernia repair		1		0		0
Elective trans-oesophageal echo		0		0		1
Gallstone pancreatitis		0		2		0
Gallstones		0		1		0
Gastritis		0		1		0
Pancreatitis		1		0		0
PR bleeding		0		1		0
Small bowel obstruction		0		1		0
Typhoid		1		0		0
**Gynaecological**	**0**	**0**	**1**	**1**	**0**	**0**
Elective hysteroscopy		0		1		0
**Musculoskeletal**	**4**	**4**	**6**	**6**	**3**	**3**
Arthritis		0		1		0
Elective toe fusion		1		0		0
Fracture - clavicle		0		0		1
Fracture – humerus		1		0		0
Fracture – NOF		0		2		1
Fracture – rib		0		1		0
Fracture – wrist		1		0		0
Lumbar 3-4 disc prolapse		1		0		0
Muscle spasm leg/back		0		1		0
Prosthetic hip replacement		0		1		0

SAE Event	RT		EULT		UC	
	**No of participants experienced event**	**Total number of events**	**No of participants experienced event**	**Total number of events**	**No of participants experienced event**	**Total number of events**
Worsening rheumatoid arthritis		0		0		1
**Neurological**	**14**	**14**	**5**	**5**	**7**	**7**
Decompensation of stroke		0		1		1
Elective cranioplasty		1		0		0
Headache due to intracranial air from cranioplasty		1		0		0
Likely new stroke		0		0		1
New stroke		2		1		1
Non-convulsive status epilepticus		1		0		0
Seizure		8		3		3
Traumatic left subdural haemotoma		1		0		0
Worsening stroke symptoms from focal seizure		0		0		1
**Respiratory**	**4**	**4**	**3**	**3**	**6**	**7**
Bronchitis		0		1		0
Elective admission for investigation of sleep apnoea		1		0		0
Influenza		0		0		1
Parapneumonic effusion		0		0		1
Pneumonia		2		0		4
Pulmonary emboli		1		2		0
Pulmonary oedema		0		0		1
**Urinary tract**	**3**	**3**	**3**	**4**	**3**	**3**
Elective biopsy of bladder lesion		0		0		1
Epididymo-orchitis		0		0		1
Hyperkaleamia and renal acidosis		0		1		0
Urinary retention		0		1		1
UTI/ urosepsis		3		2		0
**Miscellaneous**	**8**	**8**	**8**	**8**	**5**	**6**
Abdominal pain (cause unknown)		1		0		0
Acute kidney injury (cause unknown)		1		0		0
Deep vein thrombosis and chest infection		0		1		0
Diarrhoea (cause unknown)		0		1		1
Diarrhoea and acute kidney injury (cause unknown)		0		0		1
Diarrhoea and nausea secondary to Pembrolizumab		0		0		1
Elective axillary lipoma excision		0		0		1
Infected cranioplasty		0		0		1
Fall (mechanical)		1		0		0
Fall (possible post stroke seizure)		0		1		0
Head injury		1		0		0
Infected foot following injury		0		1		0
Intoxication and pneumonia		0		1		0
Likely viral illness		1		0		0
Pneumonia and seizure		0		0		1

SAE Event	RT		EULT		UC	
	No of participants experienced event	Total number of events	No of participants experienced event	Total number of events	No of participants experienced event	Total number of events
Pyrexia (cause unknown)		1		0		0
Scald/ burn		0		1		0
Transient difficulty walking (cause unknown)		0		1		0
Urosepsis and infective endocarditis		1		0		0
Vertigo		0		1		0
Worsening stroke symptoms (cause unknown)		1		0		0

Note that where SAE reports documented more than one clinical event (for example Intoxication and pneumonia), the main event/reason for hospitalisation is the event summarised. Where the main event appeared unclear, adjudication took place to agree the event to summarise.

Supplementary Table 9: **Number of non-serious adverse events (AEs) per patient**

**Number of AEs per participant**	**3 months: Number of participants**			**6 months: Number of participants**		
	**RT n= 233**	**EULT n = 236**	**UC n = 207**	**RT n = 223**	**EULT n=222**	**UC n = 190**
**0**	195 (83.7%)	199 (84.3%)	172 (83.1%)	181 (81.2%)	176 (79.3%)	163 (85.8%)
**1**	33 (14.2%)	34 (14.4%)	32 (15.5%)	40 (17.9%)	41 (18.5)	23 (12.1%)
**2**	3 (1.3 %)	3 (1.3%)	3 (1.4%)	2 (0.9%)	3 (1.4%)	4 (2.1%)
**3**	1 (0.4%)	0 (0%)	0 (0%)	0 (0%)	2(0.9%)	0 (0%)
**4**	1 (0.4%)	0 (0%)	0 (0%)	0 (0%)	0 (0%)	0 (0%)
**Total events**	46	40	38	44	53	31

Data are n (%)

Supplementary Table 10: **Summarised non-serious adverse events reported at the three month assessment**

**AE Event (3 month)**	**RT**		**EULT**		**UC**	
	**No of participants experienced event**	**Total number of events**	**No of participants experienced event**	**Total number of events**	**No of participants experienced event**	**Total number of events**
**Cardiovascular**	**0**	**0**	**0**	**0**	**2**	**2**
Heart palpitations		0		0		1
Pacemaker fitting		0		0		1
**Dental**	**1**	**1**	**0**	**0**	**0**	**0**
Tooth infection		1		0		0
**Dermatological**	**1**	**1**	**0**	**0**	**3**	**3**
Blisters on foot		0		0		1
Cyst on leg		0		0		1
Eczema		1		0		0
Leg ulcers		0		0		1
**Endocrine**	**2**	**2**	**2**	**2**	**1**	**1**
Diabetes mellitus		2		1		1
Hyperglycaemia		0		1		0
**Ear, Nose and Throat**	**1**	**1**	**0**	**0**	**2**	**2**
Ear infection		1		0		0
Oral thrush		0		0		1
Possible ear infection		0		0		1
**Eye**	**0**	**0**	**1**	**1**	**1**	**1**
Problems with eyes		0		1		0
Stye removal		0		0		1
**Gastrointestinal**	**3**	**3**	**5**	**5**	**2**	**2**
Abdominal pain and diarrhoea		0		1		0
Abnormal liver function		0		1		0
Constipation		1		0		0
Diarrhoea		0		0		2
Hernia		0		2		0
Infection at PEG		0		1		0
Reflux		1		0		0
Scope for assessment of banding		1		0		0
**Gynaecological**	**0**	**0**	**1**	**1**	**0**	**0**
Fibroid		0		1		0
**Musculoskeletal**	**9**	**9**	**5**	**6**	**5**	**5**
Arthritis - knee		0		0		1
Foot problems		1		0		0

AE Event (3 month)	RT		EULT		UC	
	**No of participants experienced event**	**Total number of events**	**No of participants experienced event**	**Total number of events**	**No of participants experienced event**	**Total number of events**
Fracture – ankle		0		1		0
Fracture – foot		0		1		0
Fracture – shoulder		1		1		0
Frozen shoulder		3		1		0
Osteoporosis		1		0		0
Podiatry referral		1		0		0
Possible arthritis		0		0		1
Shoulder pain		2		1		1
Tendonitis in arm		0		0		1
Trapped nerves in shoulder		0		0		1
Upper limb pain		0		1		0
**Neurological**	**1**	**1**	**5**	**5**	**5**	**5**
Memory loss		0		0		1
Neuropathic pain		0		1		0
Seizure (s)		1		4		4
**Psychiatric**	**2**	**2**	**3**	**3**	**2**	**2**
Depression		2		3		1
Panic attacks		0		0		1
**Respiratory**	**3**	**3**	**6**	**6**	**2**	**2**
Chest infection		2		5		2
Possible new diagnosis of COPD		0		1		0
Cough		1		0		0
**Urinary tract**	**3**	**3**	**1**	**1**	**5**	**5**
Bladder infections		0		0		1
Haematuria		1		0		0
Incontinence		0		0		2
Urinary frequency		0		0		1
Urinary retention		1		0		0
Urinary tract infection		1		1		0
Water retention		0		0		1
**Miscellaneous**	**19**	**20**	**10**	**10**	**8**	**8**
Abscess on back		1		0		0
Allergic reaction		0		1		0
Blackout		1		0		0
Bone flap reinserted (on head)		1		0		0
Commenced on new medication for blood pressure and bladder control		1		0		0
Change in drugs						
Curling toes		0		0		1
Dog bite		1		0		0
Double vision		0		0		1

**AE Event (3 month)**	**RT**		**EULT**		**UC**	
	**No of participants experienced event**	**Total number of events**	**No of participants experienced event**	**Total number of events**	**No of participants experienced event**	**Total number of events**
Drug side effects		1		0		0
Fall		2		1		0
High cholesterol		6		2		2
Joint/muscle/back pain		0		0		1
Lightheaded		2		3		0
No details provided		0		1		0
Swallowing problems		0		0		1
Swelling of foot		0		1		1
Swelling of legs		0		0		1
Swollen ankles		1		0		0
Toenail removal		2		0		0
Vertigo		1		0		0
		0		1		0

Supplementary Table 11: **Summarised non-serious adverse events reported at the six month assessment**

**AE Event (6 month)**	**RT**		**EULT**		**UC**	
	**No of participants experienced event**	**Total number of events**	**No of participants experienced event**	**Total number of events**	**No of participants experienced event**	**Total number of events**
**Cardiovascular**	**2**	**2**	**0**	**0**	**2**	**2**
High blood pressure		0		0		1
Hypotension		2		0		0
Palpitations		0		0		1
**Dermatological**	**2**	**2**	**6**	**6**	**2**	**2**
Eczema		0		1		0
Forehead lesion (cause unknown)		0		0		1
Itch		1		1		0
Infected toe		0		0		0
Leg rash		0		1		0
Leg ulcers		0		2		0
Lesion on face removed		0		1		0
Rash		1		0		1
**Ear**	**0**	**0**	**2**	**2**	**0**	**0**
Ear pain		0		1		0
Ears syringed		0		1		0
**Endocrine**	**1**	**1**	**2**	**2**	**0**	**0**
Diabetes mellitus		1		0		0
Menopause symptoms		0		1		0
Thyroid eye disease		0		1		0
**Ear, Nose and Throat**	**1**	**1**	**1**	**1**	**0**	**0**
Oral thrush		0		1		0
Potential throat cancer		1		0		0
**Eye**	**0**	**0**	**0**	**0**	**2**	**2**
Cataracts		0		0		1
Ophthalmologist review – no further information		0		0		1
**Gastrointestinal**	**5**	**5**	**3**	**3**	**2**	**2**
Constipation		1		0		0
Diarrhoea		0		0		1
Diarrhoea and vomiting		0		1		0
Gastroscopy		1		0		0
Haemorrhoids		1		0		0
Hernia		1		1		0
Irritable bowel syndrome		0		0		1
Piles		1		0		0
Rectal bleeding		0		1		0

AE Event (6 month)	RT		EULT		UC	
	**No of participants experienced event**	**Total number of events**	**No of participants experienced event**	**Total number of events**	**No of participants experienced event**	**Total number of events**
**Haematological**	**1**	**1**	**1**	**1**	0	**0**
Low blood count		1		1		0
**Musculoskeletal**	**5**	**5**	**7**	**7**	**5**	**5**
Arthritis		1		0		1
Fracture - fingers		1		1		0
Fracture - foot		1		0		1
Fracture - humerus		0		1		0
Fracture - knuckles		0		1		0
Fracture – leg		0		0		1
Fracture - rib		0		1		0
Fracture - wrist		1		0		0
Frozen shoulder		0		2		0
Injured hand in car door		1		0		0
Orthotic appointments		0		0		1
Polymyalgia		0		1		0
Pre-op assessment for knee replacement		0		0		1
**Neurological**	**5**	**5**	**5**	**5**	**4**	**4**
Foot drop		1		0		0
Migraine		0		1		0
Nerve impingement		0		1		0
Neuropathy		0		0		1
Pins and needles in affected arm		1		0		0
Seizure (s)		3		3		3
**Psychiatric**	**2**	**2**	**2**	**2**	**1**	**1**
Depression		1		1		0
Panic attacks		0		1		0
Psychiatric assessment – no further information		1		0		0
Stress		0		0		1
**Respiratory**	**2**	**2**	**6**	**6**	**1**	**1**
Asbestosis		1		0		0
Chest infection		1		1		0
COPD		0		2		0
Cough		0		0		1
Coughing blood		0		1		0
Pneumonia		0		1		0
Shortness of breath		0		1		0
**Urinary tract**	**3**	**3**	**4**	**4**	**0**	**0**
Incontinence		1		0		0
Urinary tract infection		2		4		0

AE Event (6 month)	RT		EULT		UC	
	**No of participants experienced event**	**Total number of events**	**No of participants experienced event**	**Total number of events**	**No of participants experienced event**	**Total number of events**
**Miscellaneous**	**15**	**15**	**13**	**14**	**11**	**12**
Bloating and uncomfortable		0		0		1
Chest pain (no cause documented)		1		0		0
Collapse		0		1		0
Cut finger		1		0		0
Dizziness		0		1		0
Double vision		1		0		0
Drug side effects		1		0		0
Fall		3		2		4
Headaches		1		0		0
Head injury		1		1		0
Ingrowing toenail		2		0		1
Joint/muscle/back pain		2		6		5
No details given		0		1		0
Restless leg syndrome		0		1		0
Swallowing problems		1		0		1
Swelling of legs		0		1		0
Unwell (no further details)		1		0		0

***Supplementary Figure 1:* Pre-specified subgroup analyses between mean Action Research Arm Test (ARAT) score at three months and time since stroke and severity of baseline upper limb functional limitation in the intention to treat population.**

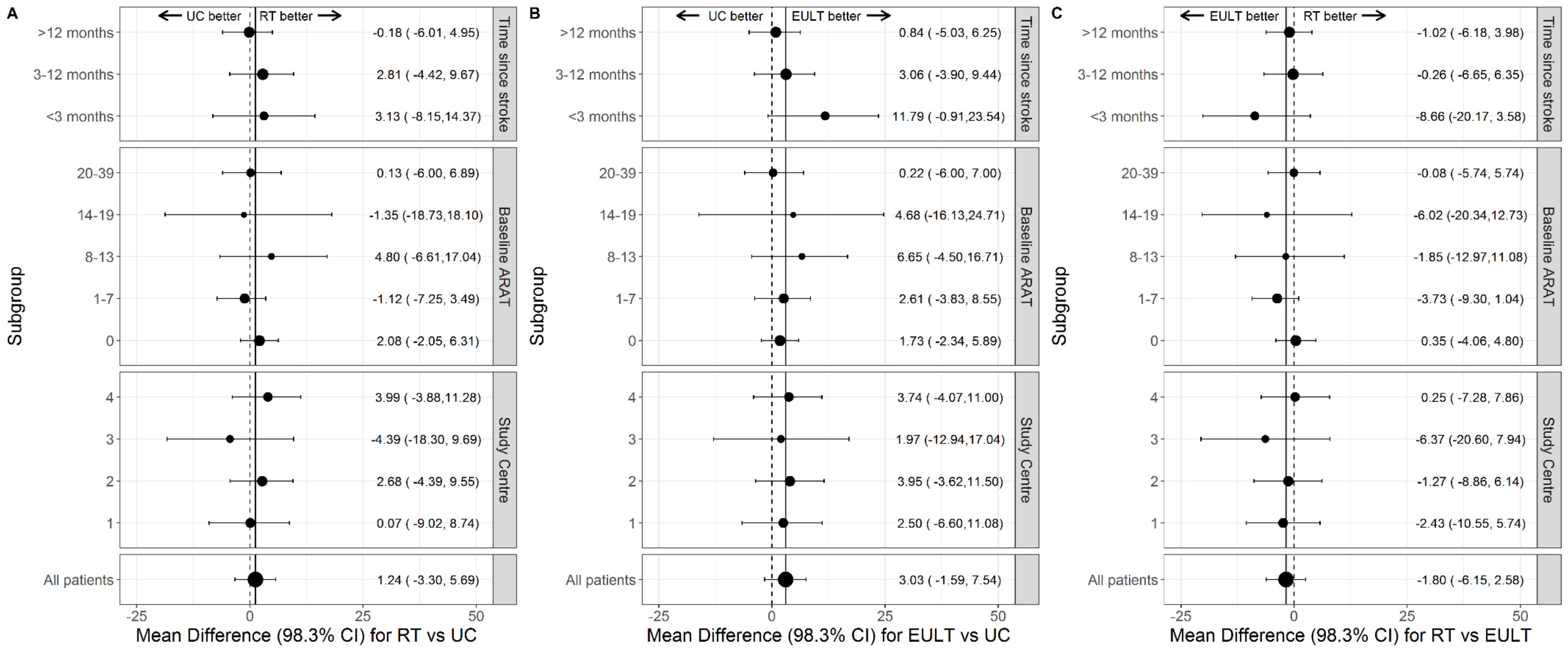

Unadjusted mean differences for the ARAT score at three months are presented with 98⅓% CI for the pre-specified subgroups of time since stroke (<3 months, 3-12 months, >12 months), baseline ARAT score (0,1-7,8-13,14-19,20-39) and centre for RT compared to UC (A), EULT compared to UC (B) and RT compared to EULT (C). The vertical solid line is the unadjusted mean difference for all participants and as all confidence intervals cross this line there is no evidence of an effect of the subgroups on the mean ARAT score at three months. The vertical dashed line is at a mean difference of zero. The points representing the unadjusted mean difference are proportional to the sample size therefore the smaller the point the smaller the sample size.

Supplementary Table 12: **Visual Analogue Scale (VAS) and utility scores before multiple imputation at all time-points**

**Utility Scores**	**RT (n= 257)**			**EULT (n=259)**			**UC (n=254)**		
	**Mean (SD)**	**Median [IQR]**	**n**	**Mean (SD)**	**Median [IQR]**	**n**	**Mean (SD)**	**Median [IQR]**	**n**
Baseline EQ-5D-5L	0.36 (0.26)	0.38 [0.17, 0.56]	254	0.39 (0.25)	0.41 [0.20, 0.57]	259	0.37 (0.26)	0.39 [0.18, 0.58]	254
3 months EQ-5D-5L	0.45 (0.27)	0.51 [0.26, 0.67]	232	0.48 (0.24)	0.55[0.32, 0.67]	236	0.42 (0.29)	0.42 [0.21, 0.64]	207
6 months EQ-5D-5L	0.46 (0.29)	0.54 [0.22, 0.71]	223	0.50 (0.27)	0.57 [0.32, 0.72]	222	0.46 (0.27)	0.53 [0.25, 0.66]	190
Baseline VAS	55.25 (21.65)	50.00 [40.00, 70.00]	254	54.58 (20.34)	50.00 [40.00, 70.00]	258	54.97 (20.52)	55.00 [40.00, 70.00]	253
3 months VAS	60.66 (20.38)	60.00 [50.00, 75.00]	233	61.49 (19.90)	60.00 [50.00, 80.00]	235	57.84 (21.81)	60.00 [45.00, 75.00]	207
6 months VAS	63.05 (20.83)	65.00 [50.00, 80.00]	223	61.88 (20.99)	65.00 [50.00, 75.00]	221	60.75 (21.36)	60.00 [50.00, 75.00]	189

Data are mean (SD), median [IQR] VAS = Visual Analogue Scale

Supplementary Table 13: **Missing information analysis for variables included in base-case adjusted analysis**

	**RT**		**EULT**		**UC**		**Total**	
	**Included (n)**	***Missing (n)***	**Included (n)**	***Missing (n)***	**Included (n)**	***Missing (n)***	**Included (n)**	***Missing (n)***
**Total costs**	257	*0*	259	*0*	178	*76*	694	*76*
**MI QALYs**	254	*3*	259	*0*	254	*0*	767	*3*
**Site**	257	*0*	259	*0*	254	*0*	770	*0*
**Baseline ARAT**	256	*1*	259	*0*	254	*0*	769	*1*
**Time since stroke**	257	*0*	259	*0*	254	*0*	770	*0*
**Baseline costs**	248	*9*	254	*5*	240	*14*	742	*28*
**Baseline utility score**	254	*3*	259	*0*	254	*0*	769	*3*

Supplementary Table 14: **Total cost per participant at six months**

**Resource use (mean costs per patient)**	**RT**		**EULT**		**UC**	
	**n**	**Mean (sd)**	**n**	**Mean (sd)**	**n**	**Mean (sd)**
Primary care costs and community based health care	213	743 (1,031)	215	777 (1,264)	177	1,078 (1,813)
Social care	213	1,410 (3,146)	216	1,541 (3,943)	178	1,890 (4,281)
Secondary care	213	733 (2,247)	216	988 (4,486)	178	668 (1,880)
Medication costs	157	149 (302)	162	154 (273)	126	198 (347)
Other NHS and social services	11	727 (983)	13	790 (946)	9	307 (406)
Deceased participants	1	0 (0)	3	13,953 (4,516)	0	0
Intervention costs	257	2,872 (0)	259	1,399 (0)	-	-
Total average cost	257	5,387 (4,054)	259	4,451 (6,033)	178	3,785 (5,437)

Data are mean (SD)

RATULS protocol amendments:

Amendments made prior to publication of the protocol:

1. Modification of invitation procedure of potential participants by letter (from both primary and secondary care)

When the trial commenced, potential participants invited by letter were asked to telephone their local trial team if they would like more information. The response rate was low and it was suggested that potential participants may prefer to respond in writing in the first instance. It was also suggested that the information contained in the invitation letter was too onerous. Therefore, a reply slip was included in the letters for potential participants to express an interest in the trial in writing. In addition, a short summary RATULS leaflet was also included in with sent letters. Furthermore, we introduced the option of re-contacting potential participants who do not respond to the initial invitation letter.

2. Introduction of reminders to complete the trial arm rehabilitation logs

The original trial protocol did not include any reminders to complete the arm rehabilitation logs. Text message reminders were introduced to try and improve completion rate.

3. Provision of trial newsletters

The trial was designed to include 10 % attrition. When it was realised that the trial attrition was higher than anticipated it was suggested that we contact the participants more regularly to maximise retention. Newsletters were mailed to participants monthly.

4. Modification of the parallel process evaluation

Amendments made since publication of the protocol:

1. Revision of recruitment target

As discussed in the main paper, when the trial commenced, the target sample size was 720 which included inflation for 10 % attrition. Unfortunately, despite efforts to maximise retention, the drop out from the trial higher than 10 %. To achieve the follow up for 216 participants in each randomisation group (648 total) to detect a difference between the trial randomisation groups the target sample size was inflated to 762 participants to allow for 15 % attrition from the trial.
